# Collaborative 3D Scene Reconstruction in Large Outdoor Environments Using a Fleet of Mobile Ground Robots

**DOI:** 10.3390/s23010375

**Published:** 2022-12-29

**Authors:** John Lewis, Pedro U. Lima, Meysam Basiri

**Affiliations:** Institute for Systems and Robotics, Instituto Superior Tecnico, University of Lisbon, 1049-001 Lisbon, Portugal

**Keywords:** scene reconstruction, cooperative mapping, point cloud registration, multi-robot system, 3D mapping, communication constraint

## Abstract

Teams of mobile robots can be employed in many outdoor applications, such as precision agriculture, search and rescue, and industrial inspection, allowing an efficient and robust exploration of large areas and enhancing the operators’ situational awareness. In this context, this paper describes an active and decentralized framework for the collaborative 3D mapping of large outdoor areas using a team of mobile ground robots under limited communication range and bandwidth. A real-time method is proposed that allows the sharing and registration of individual local maps, obtained from 3D LiDAR measurements, to build a global representation of the environment. A conditional peer-to-peer communication strategy is used to share information over long-range and short-range distances while considering the bandwidth constraints. Results from both real-world and simulated experiments, executed in an actual solar power plant and in its digital twin representation, demonstrate the reliability and efficiency of the proposed decentralized framework for such large outdoor operations.

## 1. Introduction

The use of outdoor mobile robots for real-world applications, such as search and rescue [1,2], logistics [3], agriculture [4], industrial inspection [5], surveillance and maintenance [6,7], have increased rapidly over the past several years. This is due to the capabilities of mobile robots to assist humans in dangerous, repetitive or time-consuming tasks. A successful robot navigation for such applications relies primarily on three aspects: mapping, localization, and trajectory planning. Robotic mapping generates a map by deciphering the spatial information of the environment acquired through the robot’s sensors. Commonly, mapping is carried out first to understand the environment and enhance the subsequent localization and motion planning tasks. However, for many applications, mapping must be executed frequently to continuously acquire a complete situational awareness and to support reasoning and decision making in dynamic environments.

Many outdoor robotic automation applications, such as solar farm inspection and maintenance [8,9,10], disaster response [11,12,13], agriculture [14] and city re-planning [15,16] need to cover very large areas of 1–40 acres. Traversing such expansive environments with a single mobile robot is very time-consuming or even impractical. In addition, conventional localization methods based only on GPS, odometry and IMU are not always reliable for such long-range operations. The uneven, rough, and unstructured nature of rural environments, such as in solar farms and disaster-struck regions, introduce additional localization errors. In such scenarios, a multi-robot system can be a suitable alternative to obtain full coverage of the area and execute tasks in a collaborative manner, resulting in a more complete and time-efficient solution. In regards to mapping, a multi-robot system can rapidly explore the environment in parallel and from different angles, to generate more accurate maps in less time [17] and to enhance the localization accuracy in challenging environments.

In general, a multi-robot mapping framework will require three main elements:

1. A mission-planning unit to coordinate robots to explore the environment.
2. A communication policy to share the map generated by each robot.
3. A matching and merging method to integrate individual maps into a global map.

In practice, mapping large outdoor 3D environments with a team of mobile robots is challenging due to the communication limitations and the high volume of sensor data that need to be shared and processed. High-capacity wireless communication routers commonly available to robots, such as Wi-Fi modules, typically have a limited range of about 90 m or less in open outdoor environments. On the other hand, long-range wireless devices, such as the Xbee-Pro RF module, can provide a large coverage of up to several kilometers but have a very limited bandwidth of about 200 kbs, which is not suitable for sharing high volumes of sensor data. Furthermore, the employment of 4G mobile technologies is not always possible due to the lack of coverage in many rural areas or in disaster-response scenarios. Hence, it is essential to consider such communication constraints while developing a multi-agent mapping algorithm.

In this work, we present an online, fully distributed and active framework for a team of mobile ground robots, equipped with 3D LiDAR sensors, for mapping and situational awareness in large outdoor environments. We develop our solution specifically for the application of inspection and surveillance of large multi megawatt solar plants, while considering the strict communication constraints that exist in terms of range and bandwidth in the commonly available wireless technologies. However, the proposed framework can be applied to many other outdoor exploration problems, such as search and rescue or precision agriculture. The outline of the paper is as follows: Section 2 provides the literature review on 3D mapping and localization methods and discusses the various techniques and limitations of multi-agent cooperative mapping and point cloud registration. The proposed distributed multi-agent framework is discussed in Section 3. The experiments and results are presented in Section 4. Finally, Section 5 concludes the findings and pitches possible improvements to the proposed method.

## 2. Literature Review

Over the past decade, 3D sensors have emerged as revolutionary data acquisition devices. In robotics, 3D sensory information has been used for mapping, localization, obstacle avoidance, and scene recognition. Omnidirectional LiDARs [18,19,20,21], RGBD cameras [22,23,24,25], and uni-directional LiDARs [26,27,28] have found applications in the field. Three-dimensional sensor-based algorithms, such as LOAM [18,19,21], have become the norm for an out-of-the-box simultaneous localization and mapping algorithm. However, the computational complexity of 3D algorithms and the size of 3D sensor data make it challenging to achieve scalability. Due to these reasons, SLAM (simultaneous localization and mapping) algorithms [29,30,31] are not commonly preferred with 3D sensors, especially in large areas [32,33]. Methods of point cloud compression [34] and low-cost registration [35,36] are promising endeavors but require prior training. The considerable size of 3D data further imposes constraints on a decentralized multi-agent mapping system, making it challenging to share observations continuously. Hence, it is essential to transfer only the required features.

Point clouds represent rigid body data structures, typically generated from LiDAR sensors. The process of aligning two point clouds is called point cloud registration. The process results in a rigid body transformation matrix that aligns one point cloud in the frame of another. The registration techniques [37] are categorized into local and global registration. Global registration [38,39,40,41] is ideal when the initial transformation estimate has yet to be discovered, and is perfect when the point clouds are acquired from spatially distant frames of references. When the initial transformation is known, a quick refinement can be acquired from a local registration technique. Local registration techniques, such as iterative closest point (ICP) [42,43], normal distribution transform [44], point-to-plane [45,46,47], color-based [48,49] or class-based methods [50,51], can be computationally expensive if the initial transformation is inaccurate. For multi-agent systems, where each agent has a different frame of reference, global point cloud alignment is refined by a local point cloud registration technique.

A multi-robot system relies on a consistent network for the exchange of observations and data. If the system is not centralized, the agents rely on peer-to-peer networking. However, these networks can be classified into two subcategories: long-range and short-range networks. Long-range networks, such as low power wide area (LPWA) [52,53] and long-term evolution machine type communication (LTE-M) [54] provide networking solutions for large areas. The rural area coverage analysis [55] for sigfox (30 km at 12 kbps), lora (15 km at 290 bps–50 kbps), LTE-M (10 km at 200 kbps–1 Mbps) showcases the constraints imposed on the data size. The Xbee-PRO RF modules [56] are commonly used for outdoor robot applications allowing a long-range radio-frequency (RF) transmission that can go up to several kilometers, with a limited bandwidth of (200 kbps). Short-range communication, such as Wi-Fi (100 m at 15 Mbps), are ideal for the transfer of large size data. Thus, for a distributed multi-agent mapping system, relying on peer-to-peer 3D sensory information transfer, covering a wide area (≥1 km${}^{2}$) requires both short- and long-range communication systems.

Multi-agent SLAM poses many different challenges, such as inter-agent cooperation and communication [57], distributed sensor fusion [58] and collaborative planning [59]. These challenges are further enhanced when the sensors share large information packets, such as 3D data [58]. These challenges can be relaxed in a centralized system, assisted with short-range communication devices with high bandwidth [60,61]; however, this is not a realistic solution for large outdoor applications. In a communication-constrained environment [57,62,63,64,65], prior planning [66] to meet and share information can relieve stress on communication channels. However, these periodic communications can be challenging to realize when the area to be covered exceeds 1 km${}^{2}$, especially considering the overall energy expended.

The major contribution of this article is an end-to-end active distributed homogeneous framework for the large-scale 3D mapping of environments. We incorporate a global peer-to-peer small bandwidth long-range network along with a short-range peer-to-peer network to allow a framework that can go beyond the range limits of a Wi-Fi network. The proposed approach generates a global map in each agent’s frame and helps to localize agents within this map. Conditionally, the framework heavily filters point clouds to enable long-range transmission, which is then used for localization and mapping. This conditional approach ensures that only the necessary communication bandwidth and computation are used. This relative localization can also be used for improving path planning, exploration, and mapping. The framework is developed, to tackle the communication constraints, imposed in large-area mapping.

## 3. Methodology

A set of $N_{a}$ agents, *R*, is tasked to explore and map the environment. Each agent, $R_{i}$ ($\forall R_{i}\in R$), is equipped with a 3D LiDAR sensor, a GPS receiver, an IMU, and an odometer sensor. The LiDAR sensor has a maximum range of $L_{max}$. Considering possible GPS drifts, odometer slippage, and electromagnetic interference, each agent has an instance of an extended Kalman filter (EKF) to fuse the sensory information from GPS, IMU and odometer and obtain a more reliable estimate of the global localization $G_{i}$ in the geographic coordinate system. An instance of the LiDAR odometry and mapping (LOAM) [18] is used on each agent to locally map the surrounding environment from the $R_{i}$ perspective and to locate the agent within the map. This egocentric LOAM localization is represented in the form of an odometry message $O_{i}$, in the sub-map $M_{i}$, of agent $R_{i}$. Each agent is equipped with a short-range and a long-range wireless transceiver. The short-range transceiver is a Wi-Fi module that allows the peer-to-peer transfer of large map data, which is only activated when two agents are within distance $C_{max}$ of each other. The long-range transceiver is an RF module that can ensure long-range transfer of very small quantities of data, which is used only to share odometry, GPS or heavily down-sampled 3D data.

**Figure 1:**
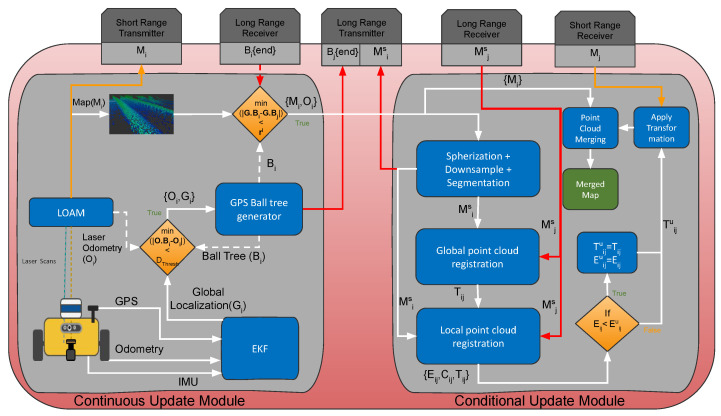
Proposed framework deployed in agent $R_{i}$.

Figure 1 portrays an instance of the proposed fast decentralized multi-agent active mapping framework, executed on agent $R_{i}$. A separate instance of the framework is executed on each agent of *R*. This ensures an active decentralized framework for multi-agent 3D mapping. The framework has two modules: a continuous update module and a conditional update module. The continuous update module is executed with every new sensory update. In this module, an instance of LOAM generates the egocentric odometry $O_{i}$ and the map $M_{i}$. Added to this, an instance of extended Kalman filter fuses the sensory data from GPS, IMU and odometry to output the global localization estimate, $G_{i}$. A ball tree generator, as explained in Section 3.1, generates a global ball tree $B_{i}$ that keeps track of $O_{i}$ and $G_{i}$ throughout time and at specified distance intervals. Whenever a new tree node is added, it is also shared with all other agents using the long-range transmitter. Minimal proximity search, detailed in Section 3.2, is used to compute the proximity of an incoming tree node from an agent $R_{j}$ with all nodes of the ball tree $B_{i}$. The conditional update module is executed when the result of the minimal proximity search is true.

Conditional update module consists of several computationally intensive processes. Spherized point cloud registration, in Section 3.3, describes down-sampling, segmentation and nearest-neighbor sampling of the $M_{i}$, to generate a spherized map ${M^{s}}_{i}$. ${M^{s}}_{i}$, which is considerably reduced in size but abundant in features, is then transmitted using the long-range transmitter to the respective agent $R_{j}$, for point cloud registration. The resultant transformation is then used for merging the complete maps once the agents are close enough to transfer the complete maps via the short-range transceiver.

### 3.1. Global Ball Tree Generator

A ball tree is a binary tree data structure, that is used for data partitioning to ensure fast data query [67]. When an agent, $R_{i}$, is initialized, a ball-tree, $B_{i}$, is instantiated with $G_{i}\left(t=0\right)$ as the root node. The node *n* of $B_{i}$ is represented as $B_{i}\left(n\right)$ and the latest node added is $B_{i}\left(end\right)$. The pair-wise distance used for constructing $B_{i}$ is the Haversine distance. The Haversine distance represents the angular distance between two points on the surface of a sphere. Ball trees with Haversine distance are shown to result in fast nearest-neighbor look-up for GPS datasets [68,69]. A node $B_{i}\left(n\right)$, inserted at time instant *T*, consists of the $G_{i}\left(t=T\right)$ and is tagged with the corresponding LOAM-odometry message $O_{i}\left(t=T\right)$. The framework continuously monitors the LOAM-odometry $O_{i}$ and iteratively calculates the distance between the current odometry $O_{i}\left(t=T\right)$ with that of the last node $B_{i}\left(end\right)$. If this distance is greater than a predefined value, $D_{thresh}$, a new node is added to $B_{i}$, with $G_{i}\left(t=T\right)$ and $O_{i}\left(t=T\right)$.

The process, called global ball-tree generator, is described in Algorithm 1, which is continuously run by each agent in *R*. Each node of the ball-tree has a global localization estimate $G_{i}$, which is mapped with the corresponding LOAM-odometry message stored at that time instant. These pair-wise data are essential to link the egocentric localization of $R_{i}$ with the global frame. Alternatively, we could georeference the point cloud, for each iteration of LOAM, which requires an accurate initial global localization estimate [70] and would be computationally costly [71,72]. The intermittent method proposed in this work eases the computational complexity. It also alleviates the dependence on a single initial estimate.
**Algorithm 1** Global ball-tree generator for agent $R_{i}$.**Input:** $G_{i}\left(t\right),O_{i}\left(t\right),D_{thresh},B_{i}$ **Initialize** $B_{i}=$ Add-Node$(G_{i}\left(t=0\right),O_{i}\left(t=0\right))$ **while** mapping **do**    Odom(Current)$=O_{i}\left(t=T\right)$    Odom(Last−Node)$=B_{i}\left(end\right)\to O$    **if** ${∥\mathrm{Odom}(Current)-\mathrm{Odom}(Last-Node)∥}_{2}\leq D_{thresh}$ **then**        $B_{i}=$Add-Node($G_{i}\left(t=T\right),O_{i}\left(t=T\right),B_{i}$)    **end if****end while** **procedure** Add-Node(G,O,B = Balltree())    B.push(G)    B(end)→O    return *B***end procedure**

### 3.2. Minimal Proximity Search

In a communication-constrained environment, it is essential to ensure that the bandwidth is used for the most vital transmissions. The process explained in this section queries for possible spatial overlaps in the global frame. Minimal proximity search transmits every new node added to the ball tree $B_{i}\left(end\right)$ and compares it with the ball trees of other agents in *R* for proximity within the global frame.

Every new node added to $B_{i}$ of agent $R_{i}$ is shared over the long-range transmitter, with the remaining agents. The minimal bandwidth required to transfer the node $B_{i}\left(end\right)$ makes it ideal for a communication-constrained environment. With no loss of generality, an agent $R_{j}$(∈R,∀i≠j) has its own instance of LOAM, EKF and global ball-tree generator, resulting in its own sub-map $M_{j}$, egocentric odometry $O_{j}$, global localization estimate $G_{j}$, and global ball-tree $B_{j}$. Agent $R_{j}$ processes the incoming node information from $R_{i}$ by carrying out a nearest-neighbor search in $B_{j}$. If the global localization estimate $G_{j}$ entry of the resultant nearest-neighbor node, $B_{j}\left(Neighbour\right)$, is within a certain threshold($r^{i}$), of $B_{i}\left(end\right)$, the node $B_{j}\left(Neighbour\right)$ is shared with $R_{i}$. This is depicted in Algorithm 2.
**Algorithm 2** Minimal proximity search by $R_{j}$.**Input:** $B_{i}\left(end\right),B_{j},r^{ij}$ **while** mapping **do**    Neighbor=$B_{j}$.NearestNeighborSearch($B_{i}\left(end\right).G$)    **if** Neighbor.distance $\leq r^{ij}$ **then**        return $B_{j}\left(Neighbor\right)$    **end if****end while**

In an effort to minimize the effect of GPS drifts, the distance threshold, $r^{i}$, is a bounded dynamic distance threshold. Equation (1) ensures that $r^{i}$ is bounded within predefined values ($r_{min}$,$r_{max}$) and proportional to the uncertainty $C_{ekf}^{i}$ of the agent $R_{i}$ EKF estimate.

(1) $$r^{i}=\left\{\begin{array}{cc} r_{max} & \mathrm{if}\;C_{ekf}^{i}\cdot r^{i}\geq r_{max}\\ r_{min} & \mathrm{if}\;C_{ekf}^{i}\cdot r^{i}\leq r_{min}\\ C_{ekf}^{i}\cdot r^{i} & \mathrm{else} \end{array}\right.$$

### 3.3. Spherized Point Cloud Registration

There exists a transformation, $T_{ij}$, that aligns $M_{j}$ with $M_{i}$ of agents $R_{j}$ and $R_{i}$. This transformation can be achieved by registering the map $M_{j}$ with the map $M_{i}$. However, the sizes of $M_{i}$ and $M_{j}$ are rapidly increasing as the $R_{i}$ and $R_{j}$ individually explore and map the environment, from their perspective. Sharing such large data over a long-range bandwidth-limited communication channel will lead to a high network latency and data loss. Hence, this section describes a strategy to only share small sampled subsets of the maps, and only for the regions that are expected to have sufficient overlapping features for registration.

With no loss in generality, let us assume that for two agents, $R_{i}$ and $R_{j}$, the minimal proximity search was successful. A successful minimal proximity search (Section 3.2) gives an assurance that, at two different time instances, the $R_{i}$ and $R_{j}$ are spatially close, in the global frame. The minimal proxy search results in two nodes, $node_{i}$ and $node_{j}$, of $B_{i}$ and $B_{j}$, that are globally close: $B_{i}\left(node_{i}\right).O$ and $B_{j}\left(node_{j}\right).O$ gives the egocentric odometry measurement of $R_{i}$ and $R_{j}$. For lucidity, we will refer to $B_{i}\left(node_{i}\right).O$ and $B_{j}\left(node_{j}\right).O$ as $L_{i}$ and $L_{j}$, respectively.

A Euclidean ball, of radius $r_{o}$, is generated in both $M_{i}$ and $M_{j}$, centered at $L_{i}$ and $L_{j}$, respectively. This method of filtering is hereby referred to as spherization. The points within the sphere are sampled and used for point cloud registration. Since they represent a fraction of the overall map, the size is considerably reduced. Added to this, the sampled map, $M_{i}^{s}$ and $M_{j}^{s}$, have features that are bound to overlap. This is because the sampled map was generated when the agents were spatially close in the global frame. Since point clouds can be considered a rigid body of particles [73], we can conclude that the $T_{ij}$ that successfully aligns $M_{i}^{s}$ with $M_{j}^{s}$ also aligns $M_{i}$ with $M_{j}$.

Spherized maps are transmitted over the long-range transmitter to the respective agents. For a seamless transmission on the constrained bandwidth channel, the spherized maps have to be less than 25 kilobytes. Thus, spherization is preceded by downsampling, ground-plane removal and outlier removal to bring down the overall size of the point cloud to the prerequisite limit. Each agent generates the spherized maps in its own frame of reference. These frames of reference will be separated by several meters, which is not ideal for a local point cloud registration algorithm. We use a global registration algorithm to align these two spherized point clouds roughly. The transformation matrix acquired from the global registration technique is then used to initialize the local point cloud registration. Local point cloud registration helps in refining the initial rough alignment. The local point cloud registration results in the transformation, $T_{ij}$, and the RMSE, $E_{ij}$, of all inlier correspondences.

The RMSE [45], in the context of point cloud registration, refers to the root mean square value between the corresponding points of the two point clouds. For $N_{c}$ correspondences, between $M_{i}^{s}$ and $M_{j}^{s}$, the RMSE for transformation, $T_{ij}$, can be calculated by Equation (2). $c_{i}$ and $c_{j}$ refer to all the correspondences in $M_{i}^{s}$ and $M_{j}^{s}$, respectively. The transformation, $T_{ij}$, that minimizes $E_{ij}$, across all executions of Algorithm 3 is chosen for the full map alignment.

(2) $$\mathrm{RMSE}=\sqrt{\frac{\sum_{n=1}^{N_{c}}{∥M_{j}^{s}\left(c_{g}^{n}\right)-T_{ij}*M_{j}^{s}\left(c_{m}^{n}\right)∥}^{2}}{N_{c}}}$$

In the proposed implementation, the global registration is carried out using RANSAC (random sample consensus) [74]. The FPFH (fast point feature histograms) feature [75], a 33-dimensional vector that encapsulates the local geometric property, for each point, is calculated. RANSAC searches for these features to make a fast and approximate alignment. For local registration, we are aware that the process can be further enhanced by sharing only the features [76,77] rather than the entire point clouds and subsequently using feature-based registration methods [45,50,51]. We could also implement a semantic mapping technique [20,21] for acquiring a segmented map before spherization. However, we use point-to-plane ICP [46] to keep the overall complexity and tunable parameters to a minimum.
**Algorithm 3** Spherized point cloud registration in agent $R_{i}$.**Input:** $M_{i},{M^{s}}_{j},B_{i},{r^{s}}_{ij}$ **if** Minimal-Proximity-Search($B_{i}\left(end\right),B_{j},{r^{s}}_{ij}$) is True **then**    ${M^{s}}_{i}$ = Spherization($M_{i}$,$B_{i}\left(end\right).O$,${r^{s}}_{ij}$)    Long-range-transmission(${M^{s}}_{i}$) -> $R_{j}$    $T_{ij}$ = Global-Point-Cloud-Registration(${M^{s}}_{i}$,${M^{s}}_{j}$)    $T_{ij}$,$E_{ij}$,$C_{ij}$ = Local-Point-Cloud-Registration(${M^{s}}_{i}$,${M^{s}}_{j}$)    **if** $E_{ij}$<${E^{u}}_{ij}$ **then**        ${E^{u}}_{ij}$=$E_{ij}$        ${T^{u}}_{ij}$=$T_{ij}$        return ${T^{u}}_{ij}$    **end if****end if** **procedure** Spherization(M,O,r)    *M* = Outlier-Removal(Ground-Plane-Removal(Downsample(*M*)))    Neighbors = *M*.NearestNeighborSearch(centre = *O*,radius = *r*)    $M^{s}$ = *M*(Neighbors)    return $M^{s}$**end procedure**

## 4. Experiments and Results

### 4.1. Real World Experiments

**Figure 2:**
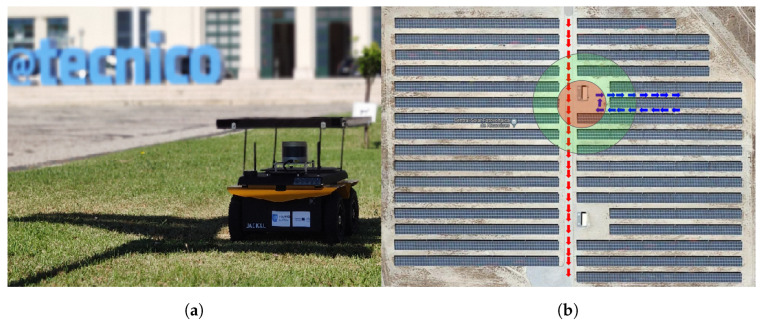
Real-world experimental setup. (**a**) Clearpath Jackal. (**b**) Path taken.

**Figure 3:**
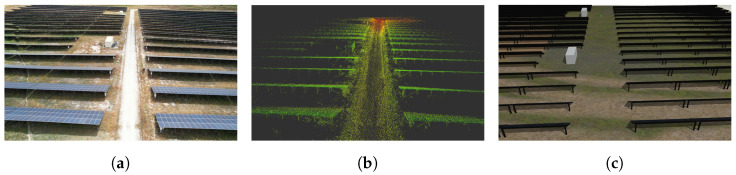
Experimental setup: Solar farm, corresponding 3D map of solar farm obtained from LOAM [18] and digital twin. (**a**) Aerial view. (**b**) 3D point cloud. (**c**) Digital Twin.

We carried out our experiments with two Jackal robots (shown in Figure 2a) (named J1 and J2), from Clearpath Robotics, on an actual solar farm (total area approximately 1 km${}^{2}$, depicted in Figure 3a). The two robots were equipped with a Velodyne Puck (VLP-16) sensor that has 16 layers of infra-red (IR) lasers, a horizontal field of view of $360^{\circ }$, a vertical field of view of $30^{\circ }$ and a speed of up to 300,000 data points per second. A Pixhawk 2.1 cube IMU and a Here+ GPS receiver were also added to each robot.

**Figure 4:**
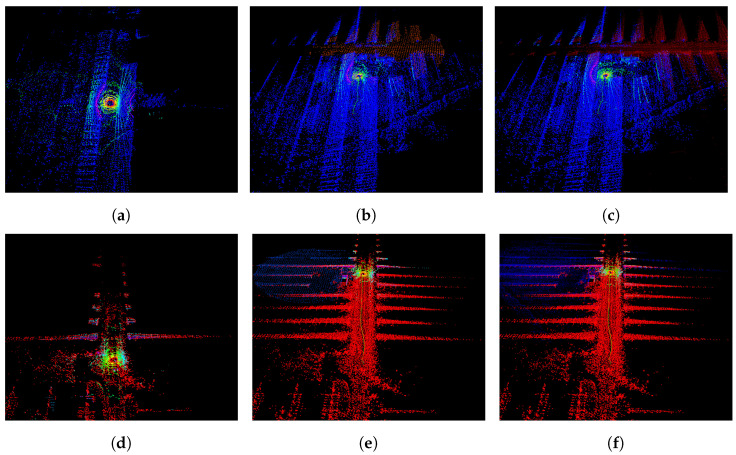
Real-world experiment—the various phases of the proposed method for agent *J*1 (**above**) and *J*2 (**below**). The blue and red points correspond to the point clouds generated by *J*1 and *J*2, respectively. (**a**,**d**) LOAM initialization; (**b**,**e**) minimal proximity search was successful, an agent receives a spherized down-sampled map from the other agent and registers this in its own map; (**c**,**f**) each agent receives the full map from the other agent, as they are within short communication range. The agent aligns the incoming map with its own map using the transformation acquired from the spherized registration.

The global path of the robots are planned beforehand to explore the regions of interest, through visiting a set of predefined GPS waypoints. The plans also include some time instances where the robots are within communication range for the sharing of map information between agents. The global paths taken by the robots are presented in Figure 2b, along with the area in which the agents were within short-range communication distance and the region that had a successful minimal proximity search outcome. The values of ($r_{max}$, $r_{min}$) were set to (20 m, 30 m). Figure 4 represents the various stages of the framework during the experiment. Each agent’s LOAM initialization (shown in Figure 4a,d) creates an ego-centric frame of reference. Once the successful minimal proximity search is achieved, the down-sampled point cloud spheres are shared between both agents. These are then registered in the respective frames of reference, as portrayed in Figure 4b,e. Finally, when the agents are close enough for short-range communication, the latest maps generated by J1 and J2 are shared and aligned, as depicted in Figure 4c,f.

### 4.2. Simulated Experiments

**Figure 5:**
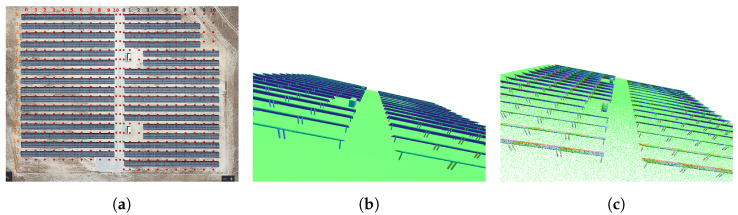
(**a**) Way points utilized for navigation, (**b**) 3D mesh of the digital twin, (**c**) 3D point cloud generated from the 3D mesh.

The simulations were carried out on a digital twin world (a 3D Gazebo model) of the actual solar farm used in the real-world experiments, as shown in Figure 3a. The simulated environment had a total area of about 1 km${}^{2}$. The digital twin was purely used for simulation purposes, to further test the collaborative 3D scene reconstruction framework with multiple agents, and was not linked to real-time sensory data from the actual solar farm. The complete ground truth point cloud was acquired by converting the 3D Gazebo mesh model (depicted in Figure 5b) to a 3D point cloud model (depicted in Figure 5c).

**Figure 6:**
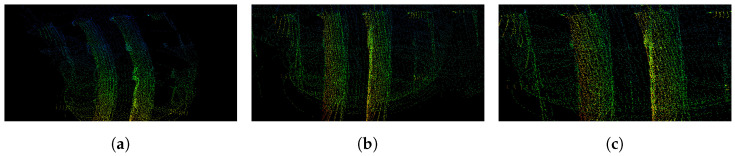
Maps, from the same viewpoint, generated with varying maximum LiDAR ranges, $L_{max}$. (**a**) $L_{max}=20$ m. (**b**) $L_{max}=40$ m. (**c**) $L_{max}=80$ m.

We initially performed a brief parameter analysis to select the values for maximum LiDAR ranges, $L_{max}$. Figure 6 details three maps, generated with three different $L_{max}$ values, by an agent following the same path in between the solar panels of the digital twin world (Figure 3c). We can note that for $L_{max}=20$ m (in Figure 6a), LOAM is unable to properly find the correspondences that are further away. Due to this, the reconstructed panels are incorrectly curved. This issue is not seen for $L_{max}=40$ m (in Figure 6b) and $L_{max}=80$ m (in Figure 6c). In an effort to keep the computational load to a minimum, $L_{max}$ was selected as 40 m for the experiments.

**Figure 7:**
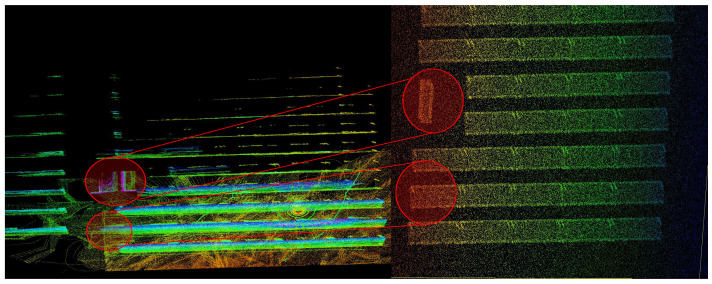
The effect of erroneous LiDAR measurements. The red circles represent the expected correspondences between the generated map and the ground truth. (**Left**): The LOAM-mapping result of an agent. (**Right**): The ground-truth 3D model.

To validate the robustness of the proposed algorithm to 3D-laser errors, we induced a Gaussian error in the simulated VLP-16 sensor. The red circles in Figure 7 map the correspondences between the ground truth and the 3D map generated by a single agent. It can be noted that, owing to LiDAR errors, there is a clear mismatch in the generated map. The resultant incorrect 3D reconstruction is evident in the encircled areas. Such reconstruction errors, across each mapping agent, is bound to make the eventual point cloud registration prone to errors. However, the cooperative framework was shown to be robust against such individual reconstruction errors and could still merge multiple maps with a good accuracy.

**Figure 8:**
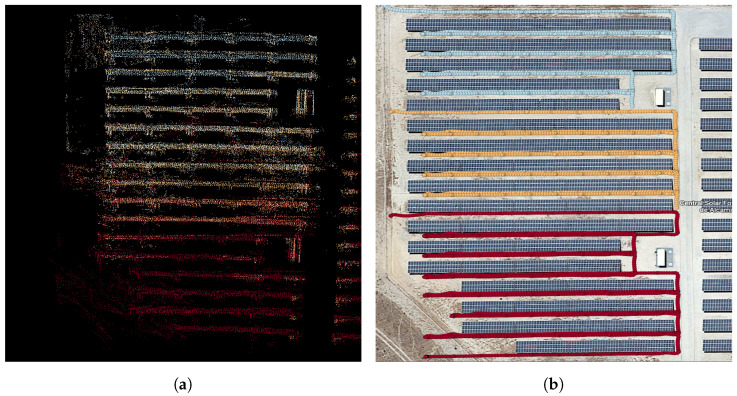
The results of an isolated iteration of simulated experiments with $N_{a}=3$. (**a**) Merged map. (**b**) Path taken.

We averaged the results over 15 simulations of varying number of UGVs ($N_{a}=2$ to 5 agents). For comparing the robustness of the proposed method to the noise in the LiDAR data and resultant LOAM mapping, we executed the experiments with different LiDAR rates, *f* of 10 hz and 5 hz. Mapping at a lower laser frequency, for the same agent speed, is relatively more error prone. Similar to the real-world experiment, navigation is carried out in between predefined waypoints, shown in Figure 5a. These waypoints are grouped as rows and divided uniformly amongst $N_{a}$. The selected path covered all possible communication conditions and allowed validation of the end-to-end functionality of the proposed framework. For $N_{a}=3$, the distribution of agents and the path taken by each agent can be seen in Figure 8b. Each color in the point cloud, shown in Figure 8a, represents the sub-map obtained by a single agent. Note that, the ground plane was removed for ease of visualization. The errors in individual LOAM-maps can be evidently seen as artifacts in Figure 8a. The framework is robust to these errors and is able to merge the maps irrespectively.

For performance analysis of the 3D scene reconstructions, we register the resultant merged map ($M_{m}$) from the simulated UGVs against the ground truth 3D model ($M_{g}$). This point cloud registration results in a transformation matrix, $T_{mg}$, and a set of corresponding points, $c_{g}$ and $c_{m}$, in $M_{g}$ and $M_{m}$, respectively.

#### 4.2.1. RMSE Analysis

**Figure 9:**
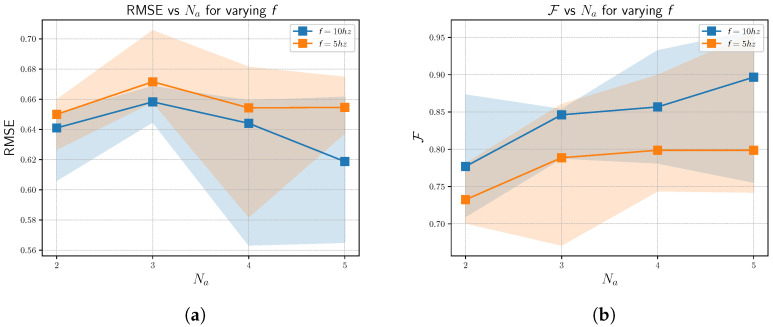
RMSE and fitness plots for varying number of agents $N_{a}$ across different rates. (**a**) RMSE. (**b**) Fitness.

Figure 9a plots the minimum–maximum–mean RMSE values for various number of agents. We can note that there is a decline in the overall RMSE values with the increase in the number of agents, $N_{a}$. The mapping error, infused by the LiDAR noise, accumulates over time. This is spread across the number of agents involved and thus the overall decline in RMSE is expected, provided the map merging is accurate. This decline in RMSE implies a successful fusion of each agent’s map.

#### 4.2.2. Fitness Analysis

Fitness( F) property of a point cloud registration gives us the overlapping area of the two point clouds. For our scenario, where the target point cloud, $M_{g}$ has $N_{g}$ points, F is given by Equation (3).

(3) $$F=\frac{N_{c}}{N_{g}}$$

Since $M_{g}$ is constant throughout the analysis, a high fitness score implies an increase in the number of correspondences. In Figure 9b, we can note that with the increase in $N_{a}$, there is a steady increase in F, (and thereby $N_{c}$), implying that the merged point clouds have more point-to-point correspondences with the $M_{g}$. This can be attributed to the successful blending of the map of each agent.

#### 4.2.3. Covariance Analysis

**Figure 10:**
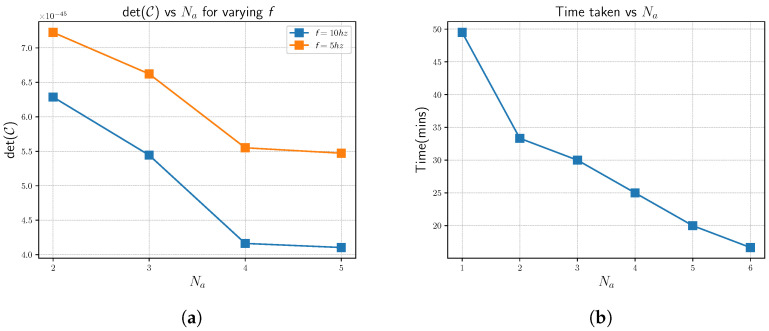
Uncertainty and time plots for varying number of agents $N_{a}$. (**a**) det(C). (**b**) Time taken.

The Fischer information matrix, I, that is acquired as a result of the point cloud registration of $M_{g}$ on $M_{m}$, characterizes the confidence in the registration process. The inverse [78] of I gives the covariance matrix, C, of the point cloud registration process [74,79,80]. C gives us the uncertainties involved in the 6 degrees of freedom. We utilize the determinant of C for our analysis of the overall uncertainty. Figure 10a showcases the healthy decline in the value of the determinant of C. This implies that the point cloud registration is more confident in its result, with the increase in $N_{a}$. The reduced covariance or the increased confidence is the result of successful map merges.

The results depicted in Figure 9 and Figure 10 further showcase the robustness of the proposed algorithm. The behavior exhibited by the agents at f=10 hz is the similar to that of f=5 hz. In other words, the agents showcase a decline in RMSE and det(C) and an increase in F with the increase in $N_{a}$. Though there is a performance degradation in f=5 hz with respect to f=10 hz, this is expected, owing to the increased mapping error from LOAM.

#### 4.2.4. Time Analysis

Trivially, the time taken to map the whole map should decline linearly with $N_{a}$. For all runs of the simulation, the homogeneous agents have the same set of waypoints to visit. Figure 10b showcases the time taken to complete the whole map. Mapping is deemed to be complete when all agents have completed Algorithm 3, with $E^{u}\leq 0.4$. This threshold gives us a reliable transformation in between maps of different agents. As is evident in Figure 10b, there is an expected decline in time taken; however, this is not linear. This is because of the time taken to achieve a successful spherized registration with low $E^{u}$.

#### 4.2.5. Density

The density, at a point(*p*) in a point cloud, is the number of points around *p*, within a sphere of radius, $r_{d}$. The density at point *p*, D(*p*), is given by Equation (4). Density can be roughly considered analogous to the resolution of an image. Thus, a denser point cloud is a more detailed point cloud.

(4) $$D\left(p\right)=\frac{\mathrm{Number}\;\mathrm{of}\;\mathrm{points}\;\mathrm{within}\;\mathrm{sphere}(\mathrm{centre}=p,\mathrm{radius}=r_{d})}{\frac{4}{3}\pi \cdot {\left(r_{d}\right)}^{3}}$$

**Figure 11:**
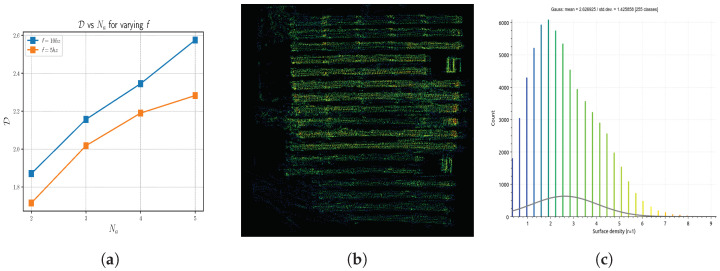
(**a**) The variation of mean density of merged map over $N_{a}$ across different rates, (**b**) the surface density distribution across the point cloud in Figure 8a, (**c**) The histogram of surface density distribution for the point cloud in Figure 8a.

For analysis, we average the density of every point in the merged point cloud [81]. The spherical radius $r_{d}$ is chosen as 1 m. The results are shown in Figure 11a. We can see a healthy increase in the average density with $N_{a}$. This is attributed to the increased overlapping areas. The overlapping areas have unique points from different agents during the merging of individual point clouds. This in-turn increases the number of points per unit area. Added to this, though trivial, it is evident that the density is higher at f=10 hz than f=5 hz. This is due to the increase rate of the LiDAR data acquisition. Figure 11b depicts the surface density distribution across each point in the point cloud, generated with 3 agents (represented in Figure 8a). Figure 11c depicts the histogram of surface density of Figure 11b. We can note that higher surface density is rare and achievable primarily in areas of overlap.

## 5. Conclusions and Future Work

This article proposes an active, distributed, homogeneous multi-agent mapping and localization framework. The distributed framework enables conditional long-range and short-range peer-to-peer communication for small and large data. The proposed method is tested on a real-world solar farm with two UGVs and its digital twin with multiple agents. The results showcase the robustness of the proposed algorithm to independent mapping errors. However, we acknowledge that using direct point cloud registration in the framework can be error-prone, with increased LiDAR errors. Additionally, a spherized registration is robust to global localization errors up to a few meters. Thus, noisy EKF estimates, due to large GPS or magnetic interference, might lead to incorrect map merges. For future work, we aim to extend the framework to a heterogeneous team of agents with a heterogeneous set of sensors. We also plan to incorporate an optimal waypoint planning module, considering the constraints in communication and each agent’s battery life. Currently, we are conducting an in-depth parameter study to understand and optimize the framework.

## Data Availability

The data presented in this study are available on request from the corresponding author.

## Abbreviations

The following abbreviations are used in this manuscript:

EKF	Extended Kalman Filter
EMI	Electromagnetic Interference
LOAM	Lidar Odometry and Mapping in Real Time
IMU	Inertial Measurement Unit

## References

[1] Basiri M., Gonçalves J., Rosa J., Bettencourt R., Vale A., Lima P. (2021). A multipurpose mobile manipulator for autonomous firefighting and construction of outdoor structures. Field Robot.

[2] Karma S., Zorba E., Pallis G., Statheropoulos G., Balta I., Mikedi K., Vamvakari J., Pappa A., Chalaris M., Xanthopoulos G. (2015). Use of unmanned vehicles in search and rescue operations in forest fires: Advantages and limitations observed in a field trial. Int. J. Disaster Risk Reduct..

[3] Limosani R., Esposito R., Manzi A., Teti G., Cavallo F., Dario P. (2018). Robotic delivery service in combined outdoor–indoor environments: Technical analysis and user evaluation. Robot. Auton. Syst..

[4] Åstrand B., Baerveldt A.J. (2002). An agricultural mobile robot with vision-based perception for mechanical weed control. Auton. Robot..

[5] Lu S., Zhang Y., Su J. (2017). Mobile robot for power substation inspection: A survey. IEEE/CAA J. Autom. Sin..

[6] Capezio F., Sgorbissa A., Zaccaria R. GPS-based localization for a surveillance UGV in outdoor areas. Proceedings of the Fifth International Workshop on Robot Motion and Control.

[7] Montambault S., Pouliot N. (2008). Design and validation of a mobile robot for power line inspection and maintenance. Proceedings of the 6th International Conference on Field and Service Robotics-FSR 2007.

[8] Akyazi Ö., Şahin E., Özsoy T., Algül M. (2019). A solar panel cleaning robot design and application. Avrupa Bilim Ve Teknoloji Dergisi.

[9] Jaradat M.A., Tauseef M., Altaf Y., Saab R., Adel H., Yousuf N., Zurigat Y.H. A fully portable robot system for cleaning solar panels. Proceedings of the 2015 10th International Symposium on Mechatronics and its Applications (ISMA).

[10] Kazem H.A., Chaichan M.T., Al-Waeli A.H., Sopian K. (2020). A review of dust accumulation and cleaning methods for solar photovoltaic systems. J. Clean. Prod..

[11] Schwarz M., Rodehutskors T., Droeschel D., Beul M., Schreiber M., Araslanov N., Ivanov I., Lenz C., Razlaw J., Schüller S. (2017). NimbRo Rescue: Solving disaster-response tasks with the mobile manipulation robot Momaro. J. Field Robot..

[12] Haynes G.C., Stager D., Stentz A., Vande Weghe J.M., Zajac B., Herman H., Kelly A., Meyhofer E., Anderson D., Bennington D. (2017). Developing a robust disaster response robot: CHIMP and the robotics challenge. J. Field Robot..

[13] Kruijff G.J.M., Kruijff-Korbayová I., Keshavdas S., Larochelle B., Janíček M., Colas F., Liu M., Pomerleau F., Siegwart R., Neerincx M.A. (2014). Designing, developing, and deploying systems to support human–robot teams in disaster response. Adv. Robot..

[14] Hajjaj S.S.H., Sahari K.S.M. Review of agriculture robotics: Practicality and feasibility. Proceedings of the 2016 IEEE International Symposium on Robotics and Intelligent Sensors (IRIS).

[15] Pfaff P., Triebel R., Stachniss C., Lamon P., Burgard W., Siegwart R. Towards mapping of cities. Proceedings of the 2007 IEEE International Conference on Robotics and Automation.

[16] Bauer A., Klasing K., Lidoris G., Mühlbauer Q., Rohrmüller F., Sosnowski S., Xu T., Kühnlenz K., Wollherr D., Buss M. (2009). The autonomous city explorer: Towards natural human-robot interaction in urban environments. Int. J. Soc. Robot..

[17] Simmons R., Apfelbaum D., Burgard W., Fox D., Moors M., Thrun S., Younes H. (2000). Coordination for multi-robot exploration and mapping. Aaai/Iaai.

[18] Zhang J., Singh S. (2014). LOAM: Lidar Odometry and Mapping in Real-time. Robotics: Science and Systems.

[19] Shan T., Englot B. Lego-loam: Lightweight and ground-optimized lidar odometry and mapping on variable terrain. Proceedings of the 2018 IEEE/RSJ International Conference on Intelligent Robots and Systems (IROS).

[20] Li L., Kong X., Zhao X., Li W., Wen F., Zhang H., Liu Y. SA-LOAM: Semantic-aided LiDAR SLAM with loop closure. Proceedings of the 2021 IEEE International Conference on Robotics and Automation (ICRA).

[21] Chen S.W., Nardari G.V., Lee E.S., Qu C., Liu X., Romero R.A.F., Kumar V. (2020). Sloam: Semantic lidar odometry and mapping for forest inventory. IEEE Robot. Autom. Lett..

[22] Yousif K., Taguchi Y., Ramalingam S. MonoRGBD-SLAM: Simultaneous localization and mapping using both monocular and RGBD cameras. Proceedings of the 2017 IEEE International Conference on Robotics and Automation (ICRA).

[23] Loianno G., Thomas J., Kumar V. Cooperative localization and mapping of MAVs using RGB-D sensors. Proceedings of the 2015 IEEE International Conference on Robotics and Automation (ICRA).

[24] Apriaskar E., Nugraha Y.P., Trilaksono B.R. Simulation of simultaneous localization and mapping using hexacopter and RGBD camera. Proceedings of the 2017 2nd International Conference on Automation, Cognitive Science, Optics, Micro Electro-Mechanical System, and Information Technology (ICACOMIT).

[25] Paton M., Kosecka J. Adaptive rgb-d localization. Proceedings of the 2012 Ninth Conference on Computer and Robot Vision.

[26] Lin J., Zhang F. Loam livox: A fast, robust, high-precision LiDAR odometry and mapping package for LiDARs of small FoV. Proceedings of the 2020 IEEE International Conference on Robotics and Automation (ICRA).

[27] Xu W., Zhang F. (2021). Fast-lio: A fast, robust lidar-inertial odometry package by tightly-coupled iterated kalman filter. IEEE Robot. Autom. Lett..

[28] Xu W., Cai Y., He D., Lin J., Zhang F. (2022). Fast-lio2: Fast direct lidar-inertial odometry. IEEE Trans. Robot..

[29] Durrant-Whyte H., Bailey T. (2006). Simultaneous localization and mapping: Part I. IEEE Robot. Autom. Mag..

[30] Bailey T., Durrant-Whyte H. (2006). Simultaneous localization and mapping (SLAM): Part II. IEEE Robot. Autom. Mag..

[31] Kim P., Chen J., Cho Y.K. (2018). SLAM-driven robotic mapping and registration of 3D point clouds. Autom. Constr..

[32] Takleh T.T.O., Bakar N.A., Rahman S.A., Hamzah R., Aziz Z. (2018). A brief survey on SLAM methods in autonomous vehicle. Int. J. Eng. Technol..

[33] Jiang Z., Zhu J., Lin Z., Li Z., Guo R. (2020). 3D mapping of outdoor environments by scan matching and motion averaging. Neurocomputing.

[34] Wiesmann L., Milioto A., Chen X., Stachniss C., Behley J. (2021). Deep compression for dense point cloud maps. IEEE Robot. Autom. Lett..

[35] Navarrete J., Viejo D., Cazorla M. (2018). Compression and registration of 3D point clouds using GMMs. Pattern Recognit. Lett..

[36] Wiesmann L., Guadagnino T., Vizzo I., Grisetti G., Behley J., Stachniss C. (2022). DCPCR: Deep Compressed Point Cloud Registration in Large-Scale Outdoor Environments. IEEE Robot. Autom. Lett..

[37] Huang X., Mei G., Zhang J., Abbas R. (2021). A comprehensive survey on point cloud registration. arXiv.

[38] Choy C., Dong W., Koltun V. Deep global registration. Proceedings of the IEEE/CVF Conference on Computer Vision and Pattern Recognition.

[39] Zhou Q.Y., Park J., Koltun V. (2016). Fast global registration. European Conference on Computer Vision.

[40] Yang H., Shi J., Carlone L. (2020). Teaser: Fast and certifiable point cloud registration. IEEE Trans. Robot..

[41] Lei H., Jiang G., Quan L. (2017). Fast descriptors and correspondence propagation for robust global point cloud registration. IEEE Trans. Image Process..

[42] Besl P.J., McKay N.D. (1992). Method for registration of 3-D shapes. Sensor Fusion IV: Control Paradigms and Data Structures.

[43] Chen Y., Medioni G. (1992). Object modelling by registration of multiple range images. Image Vis. Comput..

[44] Biber P., Straßer W. The normal distributions transform: A new approach to laser scan matching. Proceedings of the 2003 IEEE/RSJ International Conference on Intelligent Robots and Systems (IROS 2003) (Cat. No. 03CH37453).

[45] Rusinkiewicz S., Levoy M. Efficient variants of the ICP algorithm. Proceedings of the Third International Conference on 3-D Digital Imaging and Modeling.

[46] Low K.L. (2004). Linear least-squares optimization for point-to-plane icp surface registration. Chapel Hill Univ. North Carol..

[47] Park S.Y., Subbarao M. (2003). An accurate and fast point-to-plane registration technique. Pattern Recognit. Lett..

[48] Park J., Zhou Q.Y., Koltun V. Colored point cloud registration revisited. Proceedings of the IEEE International Conference on Computer Vision.

[49] Huhle B., Magnusson M., Straßer W., Lilienthal A.J. Registration of colored 3D point clouds with a kernel-based extension to the normal distributions transform. Proceedings of the 2008 IEEE International Conference on Robotics and Automation.

[50] Zaganidis A., Sun L., Duckett T., Cielniak G. (2018). Integrating deep semantic segmentation into 3-d point cloud registration. IEEE Robot. Autom. Lett..

[51] Zaganidis A., Magnusson M., Duckett T., Cielniak G. Semantic-assisted 3D normal distributions transform for scan registration in environments with limited structure. Proceedings of the 2017 IEEE/RSJ International Conference on Intelligent Robots and Systems (IROS).

[52] Raza U., Kulkarni P., Sooriyabandara M. (2017). Low power wide area networks: An overview. IEEE Commun. Surv. Tutorials.

[53] Ikpehai A., Adebisi B., Rabie K.M., Anoh K., Ande R.E., Hammoudeh M., Gacanin H., Mbanaso U.M. (2018). Low-power wide area network technologies for Internet-of-Things: A comparative review. IEEE Internet Things J..

[54] Vaezi M., Azari A., Khosravirad S.R., Shirvanimoghaddam M., Azari M.M., Chasaki D., Popovski P. (2022). Cellular, wide-area, and non-terrestrial IoT: A survey on 5G advances and the road toward 6G. IEEE Commun. Surv. Tutorials.

[55] Vejlgaard B., Lauridsen M., Nguyen H., Kovács I.Z., Mogensen P., Sorensen M. Coverage and capacity analysis of sigfox, lora, gprs, and nb-iot. Proceedings of the 2017 IEEE 85th Vehicular Technology Conference (VTC Spring).

[56] XBee RF Modules. http://www.digi.com/products/xbee-rf-solutions.

[57] Corah M., O’Meadhra C., Goel K., Michael N. (2019). Communication-efficient planning and mapping for multi-robot exploration in large environments. IEEE Robot. Autom. Lett..

[58] Xu X., Zhang L., Yang J., Cao C., Wang W., Ran Y., Tan Z., Luo M. (2022). A review of multi-sensor fusion slam systems based on 3D LIDAR. Remote Sens..

[59] Valencia R., Morta M., Andrade-Cetto J., Porta J.M. (2013). Planning reliable paths with pose SLAM. IEEE Trans. Robot..

[60] Krinkin K., Filatov A., yom Filatov A., Huletski A., Kartashov D. Evaluation of modern laser based indoor slam algorithms. Proceedings of the 2018 22nd Conference of Open Innovations Association (FRUCT).

[61] Sayed A.S., Ammar H.H., Shalaby R. Centralized multi-agent mobile robots SLAM and navigation for COVID-19 field hospitals. Proceedings of the 2020 2nd Novel Intelligent and Leading Emerging Sciences Conference (NILES.

[62] Liu T.M., Lyons D.M. (2015). Leveraging area bounds information for autonomous decentralized multi-robot exploration. Robot. Auton. Syst..

[63] Matignon L., Jeanpierre L., Mouaddib A.I. Coordinated multi-robot exploration under communication constraints using decentralized markov decision processes. Proceedings of the Twenty-Sixth AAAI Conference on Artificial Intelligence.

[64] Arkin R.C., Diaz J. Line-of-sight constrained exploration for reactive multiagent robotic teams. Proceedings of the 7th International Workshop on Advanced Motion Control.

[65] Amigoni F., Banfi J., Basilico N. (2017). Multirobot exploration of communication-restricted environments: A survey. IEEE Intell. Syst..

[66] Gao Y., Wang Y., Zhong X., Yang T., Wang M., Xu Z., Wang Y., Xu C., Gao F. (2021). Meeting-Merging-Mission: A Multi-robot Coordinate Framework for Large-Scale Communication-Limited Exploration. arXiv.

[67] Omohundro S.M. (1989). Five Balltree Construction Algorithms.

[68] Boeing G. (2018). Clustering to reduce spatial data set size. arXiv.

[69] Bhatia N. (2010). Survey of nearest neighbor techniques. arXiv.

[70] Hariz F., Souifi H., Leblanc R., Bouslimani Y., Ghribi M., Langin E., Mccarthy D. Direct Georeferencing 3D Points Cloud Map Based on SLAM and Robot Operating System. Proceedings of the 2021 IEEE International Symposium on Robotic and Sensors Environments (ROSE).

[71] Liu W., Li Z., Li Y., Sun S., Sotelo M.A. (2019). Using weighted total least squares and 3-D conformal coordinate transformation to improve the accuracy of mobile laser scanning. IEEE Trans. Geosci. Remote Sens..

[72] Janata T., Cajthaml J. (2020). Georeferencing of multi-sheet maps based on least squares with constraints—First military mapping survey maps in the area of Czechia. Appl. Sci..

[73] Yang H. (2020). A dynamical perspective on point cloud registration. arXiv.

[74] Choi S., Zhou Q.Y., Koltun V. Robust reconstruction of indoor scenes. Proceedings of the IEEE Conference on Computer Vision and Pattern Recognition.

[75] Rusu R.B., Blodow N., Beetz M. Fast point feature histograms (FPFH) for 3D registration. Proceedings of the 2009 IEEE International Conference on Robotics and Automation.

[76] Shen Z., Liang H., Lin L., Wang Z., Huang W., Yu J. (2021). Fast Ground Segmentation for 3D LiDAR Point Cloud Based on Jump-Convolution-Process. Remote Sens..

[77] Zhang F., Fang J., Wah B., Torr P. (2020). Deep fusionnet for point cloud semantic segmentation. European Conference on Computer Vision.

[78] Fujita K., Okada K., Katahira K. (2022). The Fisher Information Matrix: A Tutorial for Calculation for Decision Making Models.

[79] Pulli K. Multiview registration for large data sets. Proceedings of the Second International Conference on 3-d Digital Imaging and Modeling (Cat. No. pr00062).

[80] Barczyk M., Bonnabel S., Goulette F. (2014). Observability, Covariance and Uncertainty of ICP Scan Matching. arXiv.

[81] Maset E., Scalera L., Beinat A., Visintini D., Gasparetto A. (2022). Performance Investigation and Repeatability Assessment of a Mobile Robotic System for 3D Mapping. Robotics.

